# Assaying ADAMTS13 Activity as a Potential Prognostic Biomarker for Sinusoidal Obstruction Syndrome in Mice

**DOI:** 10.3390/ijms242216328

**Published:** 2023-11-15

**Authors:** Masakazu Saeki, Seiichi Munesue, Yuri Higashi, Ai Harashima, Ryohei Takei, Satoshi Takada, Shinichi Nakanuma, Tetsuo Ohta, Shintaro Yagi, Hidehiro Tajima, Yasuhiko Yamamoto

**Affiliations:** 1 Department of Gastroenterological Surgery, Kanazawa University Graduate School of Medical Sciences, 13-1 Takara-machi, Kanazawa 920-8641, Japan; kuariarerra7@gmail.com (M.S.); yuri.higa11@gmail.com (Y.H.); ryohei_20@hotmail.com (R.T.); n_shin@gj8.so-net.ne.jp (S.N.); ohtat@staff.kanazawa-u.ac.jp (T.O.); yagi@med.kanazawa-u.ac.jp (S.Y.); h-tajima705@dokkyomed.ac.jp (H.T.); 2 Department of Biochemistry and Molecular Vascular Biology, Kanazawa University Graduate School of Medical Sciences, 13-1 Takara-machi, Kanazawa 920-8640, Japan; smunesue@med.kanazawa-u.ac.jp (S.M.); aharashima@staff.kanazawa-u.ac.jp (A.H.); 3 Department of Gastroenterological Surgery, Dokkyo Medical University Saitama Medical Center, 2-1-50 Minami-Koshigaya, Koshigaya City 343-8555, Japan

**Keywords:** sinusoidal obstruction syndrome (SOS), a disintegrin-like metalloproteinase with thrombospondin type 1 motifs 13 (ADAMTS13), von Willebrand factor (VWF)

## Abstract

Sinusoidal obstruction syndrome (SOS) is a serious liver disorder that occurs after liver transplantation, hematopoietic stem cell transplantation, and the administration of anticancer drugs. Since SOS is a life-threatening condition that can progress to liver failure, early detection and prompt treatment are required for the survival of patients with this condition. In this study, female CD1 mice were divided into treatment and control groups after the induction of an SOS model using monocrotaline (MCT, 270 mg/kg body weight intraperitoneally). The mice were analyzed at 0, 12, 24, and 48 h after MCT administration, and blood and liver samples were collected for assays and histopathology tests. SOS was observed in the livers 12 h after MCT injection. In addition, immunohistochemical findings demonstrated CD42b-positive platelet aggregations, positive signals for von Willebrand factor (VWF), and a disintegrin-like metalloproteinase with thrombospondin type 1 motifs 13 (ADAMTS13) in the MCT-exposed liver sinusoid. Although ADAMTS13’s plasma concentrations peaked at 12 h, its enzyme activity continuously decreased by 75% at 48 h and, inversely and proportionally, concentrations in the VWF-A2 domain, in which the cleavage site of ADAMTS13 is located, increased after MCT injection. These findings suggest that the plasma concentration and activity of ADAMTS13 could be useful biomarkers for early detection and therapeutic intervention in patients with SOS.

## 1. Introduction

The liver is a main homeostatic organ, and continuous exposure of the liver to some factors leads to liver damage or injury. Liver diseases are an important and undoubtably underestimated public health problem worldwide and are known to cause a substantial level of morbidity and mortality [1]. Sinusoidal obstruction syndrome (SOS) is a serious liver injury that develops following liver transplantation or anticancer drug therapies. Clinical diagnosis of SOS has been based on the Baltimore or modified Seattle criteria, both of which assess common signs and symptoms of SOS (e.g., hyperbilirubinemia, ascites, body weight gain, and hepatomegaly) [2,3]. SOS has been reported to occur in 0.3~2.3% of post-liver transplantation, 10~15% of hematopoietic stem cell transplantation, and 59% of patients receiving preoperative oxaliplatin treatment [2,3]. Although this disease is not commonly encountered, SOS has a very poor prognosis, starting with sinusoidal endothelial damage, followed by hepatocellular necrosis and fibrosis, which progress to portal hypertension and liver failure [2,3]. It is reported that patients with pre-existent liver disease are at a substantially increased risk of hepatic SOS [4]. However, biomarkers that could aid in the early detection of SOS have been largely undiscussed in the existing literature. Dissection and desquamation of hepatic sinusoidal endothelial cells have been observed in the livers of patients with SOS due to the disruption of hepatic sinusoidal endothelial cells, followed by the extravasated platelet aggregation (EPA) of sinusoidal platelets along with impaired liver function [5,6]. In liver biopsies from recipients after living-donor liver transplants, our group found the dissection and desquamation of sinusoidal endothelial cells, mainly in the pericentral venous region, followed by EPA [7].

Von Willebrand factor (VWF) is a complex and large multimeric glycoprotein that plays an essential role in regulating hemostasis [8]. VWF is known to bind the platelet membrane glycoprotein CD42b (platelet glycoprotein Iba, GPIba) [8]. VWF mediates both platelet adhesion to the subendothelial collagen matrix and platelet–platelet interactions under high shear conditions, thereby promoting the primary attachment of platelets to damaged endothelial cells and facilitating primary hemostasis [9]. In addition, VWF consists of a series of oligomers, each comprising a variable number of subunits, ranging from a minimum of two subunits to a maximum of >40 subunits [10]. These oligomers are joined together to construct VWF multimers. The ability of VWF to maintain hemostasis depends on its multimeric structure, with high-molecular-weight (HMW) multimers boasting a greater hemostatic ability [10]. Furthermore, VWF is synthesized in endothelial cells and megakaryocytes as pro-VWF subunits, which dimerize in the cellular endoplasmic reticulum and subsequently form multimers in the Golgi apparatus [11]. These VWF multimers are stored as large multimeric forms termed ‘ultra-large VWF’ (UL-VWF) in endothelial cell Weibel–Palade bodies (WPBs) and platelet alpha granules [12]. WPB exocytosis and the release of VWF multimers are triggered by two main mechanisms: (i) agonists such as histamine and thrombin, which increase cytosolic Ca^2+^ concentrations; and (ii) agonists such as epinephrine and vasopressin, which increase cyclic adenosine monophosphate levels [13].

**Figure 1:**
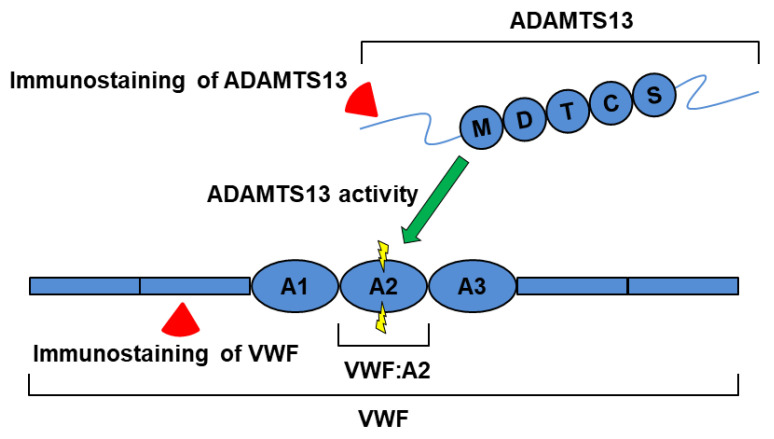
Representative schema of the interaction between ADAMTS13 and VWF. The metalloprotease ADAMTS13 is able to cleave the VWF A2 domain (green arrow), resulting in a dramatical reduction in VWF multimer formation. The ADAMTS13 C-terminal tail is known to be associated with its protease action. M, metalloproteinase domain; D, disintegrin-like domain; T, thrombospondin type 1 domain; C, cysteine-rich domain; and S, spacer domain of ADAMTS13. VWF globular domain A1 (A1) is a binding site with the platelet membrane glycoprotein CD42b (GPIbα). The A2 domain (A2) is a cleavage site recognized by ADAMTS13. The A3 domain (A3) is responsible for binding to collagen. For immunohistochemistry, we employed an ADAMTS13 antibody and a VWF antibody, which recognized the upper and lower red triangle marks, respectively, in this study.

A disintegrin-like metalloproteinase with thrombospondin type 1 motifs 13 (ADAMTS13) is a zinc metalloproteinase belonging to the ADAMTS family. ADAMTS13 is the specific cleaving protease for VWF (Figure 1). ADAMTS13 is reported to be produced by hepatic stellate cells as well as vascular endothelial cells, platelets, and glomerular epithelial cells [14,15]. It consists of a metalloproteinase (M) domain, disintegrin-like (D) domain, thrombospondin type 1 (T) domain, cysteine-rich (C) domain, and spacer (S) domain, plus seven contiguous T and two CUB (complement component Clr/Cls, Uegf, and bone morphogenic protein 1) domains, starting from the N-terminus [14] (Figure 1). Previous studies demonstrate that under physiological pH conditions, ADAMTS13 folds between T-domains, with the C-terminal region overshadowing the N-terminal region, thus suppressing its activity [16,17]. Since ADAMTS13 is responsible for moderating VWF size and function, an ADAMTS13 insufficiency can result in increased VWF levels, particularly HMW-VWF. A severe ADAMTS13 insufficiency to <10% of its normal levels or activity results in a rare life-threatening disorder called thrombotic thrombocytopenic purpura (TTP), in which an abnormal accumulation of HMW-VWF multimers results in greatly increased platelet binding and the formation of thrombi that promote microvascular occlusion [18,19].

VWF circulates in an inactive globular form upon initial release into circulation; however, it undergoes a conformational change when subjected to sufficient mechanical (‘shear’) forces [20]. Mechanical-force-dependent conformational change transforms VWF into an extended (‘elongated’) active form. This exposes critical domains involved in binding to platelets and collagen, as well as the ADAMTS13 cleavage site at the A2 domain [18] (Figure 1). ADAMTS13 regulates VWF multimer distribution and limits its prothrombotic action by cleaving these active UL-VWF multimers into smaller ones. This relationship between VWF and ADAMTS13 can be described as the VWF-ADAMTS13 axis and is indicative of vascular endothelial function [17].

ADAMTS13 has been considered as a possible biomarker for the early detection of hepatocellular carcinoma and thrombotic microangiopathy after liver transplantation [5]. Additionally, its possibility as an early detection biomarker has been discussed in ischemic heart disease and stroke [21], but not in SOS. A case report on living-donor liver transplantation showed aggregation-like VWF deposition and the expression of spot-like ADAMTS13 in liver sinusoidal vessels, with a decrease of approximately 70% in plasma ADAMTS13 activity (ADAMTS13:AC) [7]. 

We have previously reported the induction of platelet aggregation within analogous sinuses in a rat SOS model [22,23]. Considering the relationship between platelets, ADAMTS13, and VWF, we hypothesized that ADAMTS13 might be consumed to cleave VWF in SOS.

Therefore, this study evaluated the plasma levels of ADAMTS13 and VWF as biomarkers to aid in the early detection of SOS and therapeutic interventions in clinical settings, using a mouse SOS model.

## 2. Results

### 2.1. Presence of SOS and Time Course of VWF in H&E Staining and Immunohistological Staining

**Figure 2:**
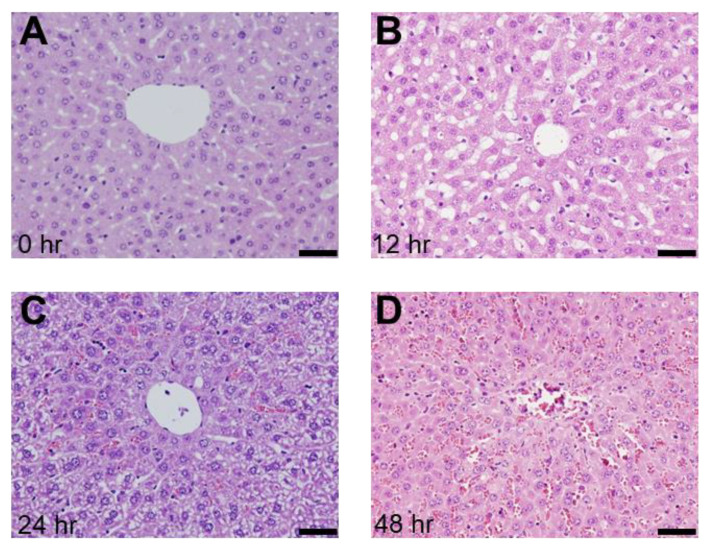
Histological findings (H&E staining) of the liver (**A**) 0 h, (**B**) 12 h, (**C**) 24 h, and (**D**) 48 h after MCT injection in the mice. Bars indicate 200 μm.

**Figure 3:**
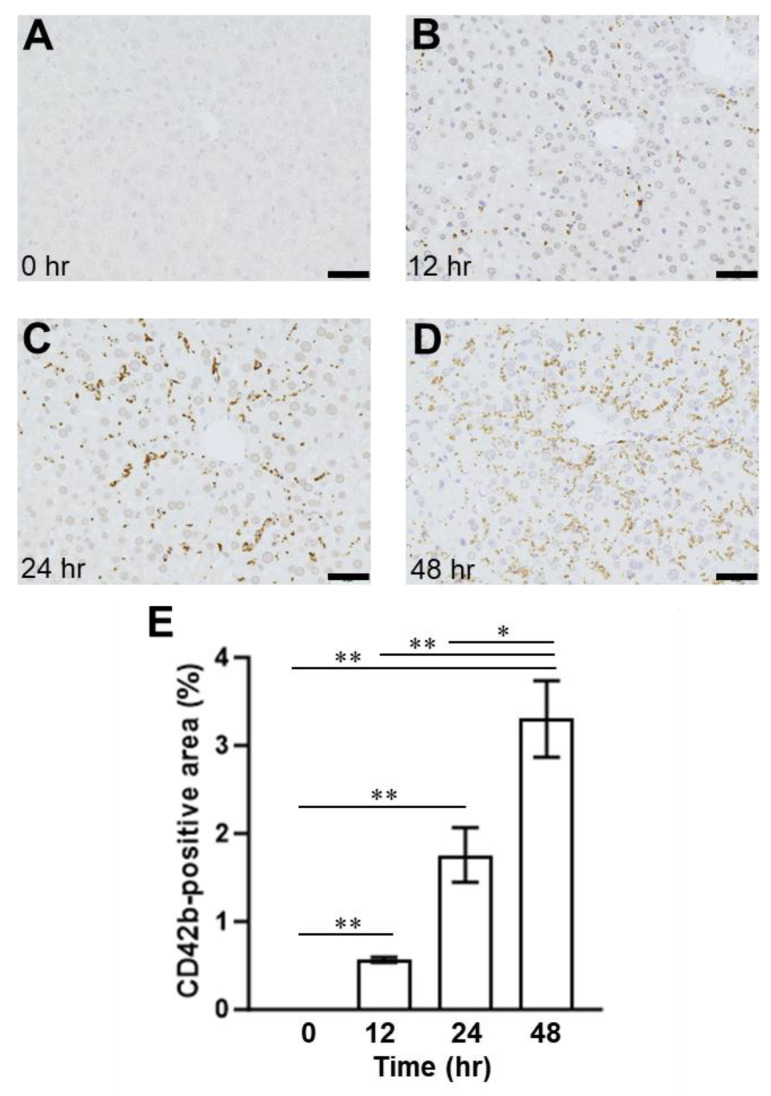
The immunohistochemical findings of CD42b (platelet glycoprotein 1bα)-positive staining in the liver (**A**) 0 h, (**B**) 12 h, (**C**) 24 h, (**D**) 48 h after MCT injection in the mice. Bars indicate 200 μm. (**E**) Quantitative evaluation of the CD42b-positive area. Data are expressed as mean ± SD (*n* = 4). **, *p* < 0.001; *, *p* < 0.05.

**Figure 4:**
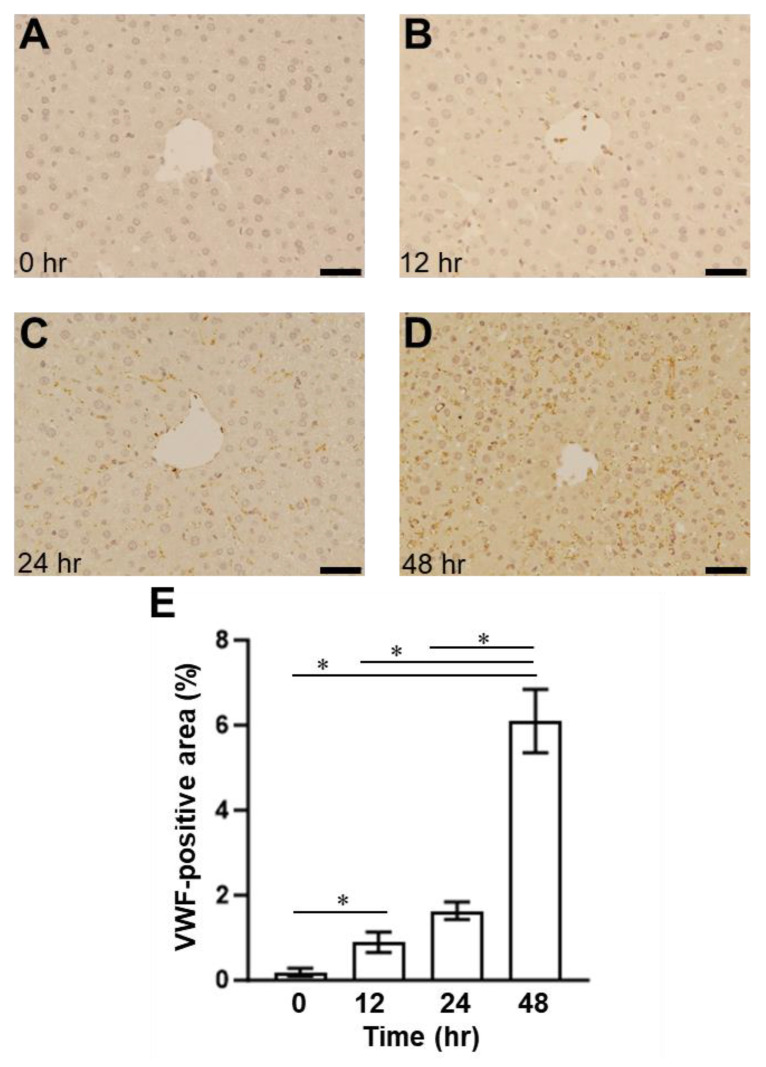
The immunohistochemical findings of VWF-positive staining in the liver (**A**) 0 h, (**B**) 12 h, (**C**) 24 h, and (**D**) 48 h after MCT injection in the mice. Bars indicate 200 μm. (**E**) Quantitative evaluation of the VWF-positive area. Data are expressed as mean ± SD (*n* = 4). *, *p* < 0.05.

To examine the relationship between VWF and ADAMTS13, we employed an SOS mouse model induced by an intraperitoneal MCT injection, as previously described [24,25]. The histological findings showed blood congestion with sinusoidal dilation and hepatocyte destruction around the central vein in the MCT-exposed liver but not in the control group (Figure 2). Congestion and hepatocyte damages were evident 24 h and 48 h after the MCT injection (Figure 2). EPA was detected in the livers of mice with induced SOS 12 h after MCT injection, using immunostaining for CD42b (PGIbα), which has a VWF binding site (Figure 3). The CD42b-positive area was time-dependently increased in the liver of MCT-injected mice during the observation period (Figure 3). In addition, we also evaluated VWF expression in the liver; VWF-positive signals increased in the liver of the MCT-injected mice in a time-dependent manner (Figure 4).

### 2.2. Plasma Concentrations of VWF in ELISA in SOS Model Mice Treated with MCT

**Figure 5:**
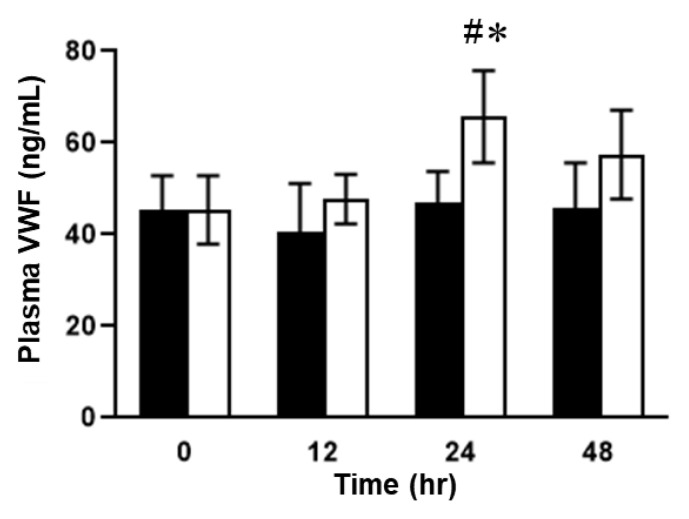
Plasma VWF concentrations. Open column, MCT-treated group; closed column, control. Data are expressed as mean ± SD (*n* = 7). #, *p* < 0.02 vs. control 0 h; *, *p* < 0.05 vs. MCT group 0 h or control 24 h.

**Figure 6:**
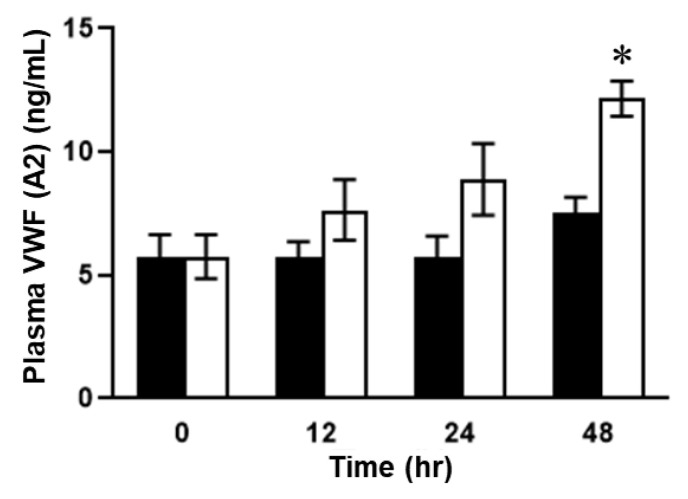
Plasma VWF (A2) concentrations. Open column, MCT-treated group; closed column, control. Data are expressed as mean ± SD (*n* = 7). Data are expressed as mean ± SD (*n* = 7). *, *p* < 0.001 vs. MCT group 0 h or control 48 h.

Next, we examined the plasma concentrations of VWF and VWF A2 domain [VWF (A2)], the cleaved site of ADAMTS13, using different ELISA systems. The plasma VWF levels of mice in the MCT-treated group were significantly upregulated 24 h after MCT administration compared to those of mice in the control group (Figure 5). After 48 h, the increased VWF levels in mice in the control group returned to baseline levels (Figure 5). In contrast, the concentrations of VWF (A2) in the plasma of the mice with SOS continued to rise during the observation period (Figure 6). There was a significant difference in plasma VWF (A2) levels at 48 h between the mice in the MCT-treated group and those in the control group (Figure 6).

### 2.3. Relationship between Plasma ADAMTS13 Levels and Immunohistological Changes in a Mouse Model of SOS

**Figure 7:**
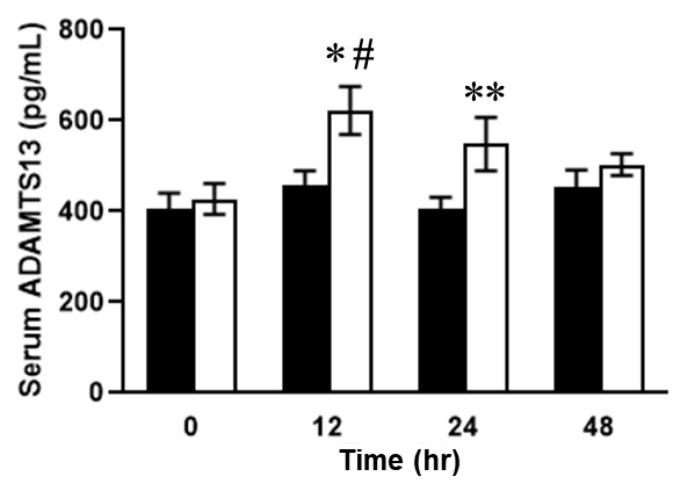
Serum ADAMTS13 concentrations. Open column, MCT-treated group; closed column, control. Data are expressed as mean ± SD (*n* = 7). *, *p* < 0.01 vs. MCT 0 h; #, *p* < 0.05, vs. control 12 h; **, *p* < 0.01 vs. control 24 h.

**Figure 8:**
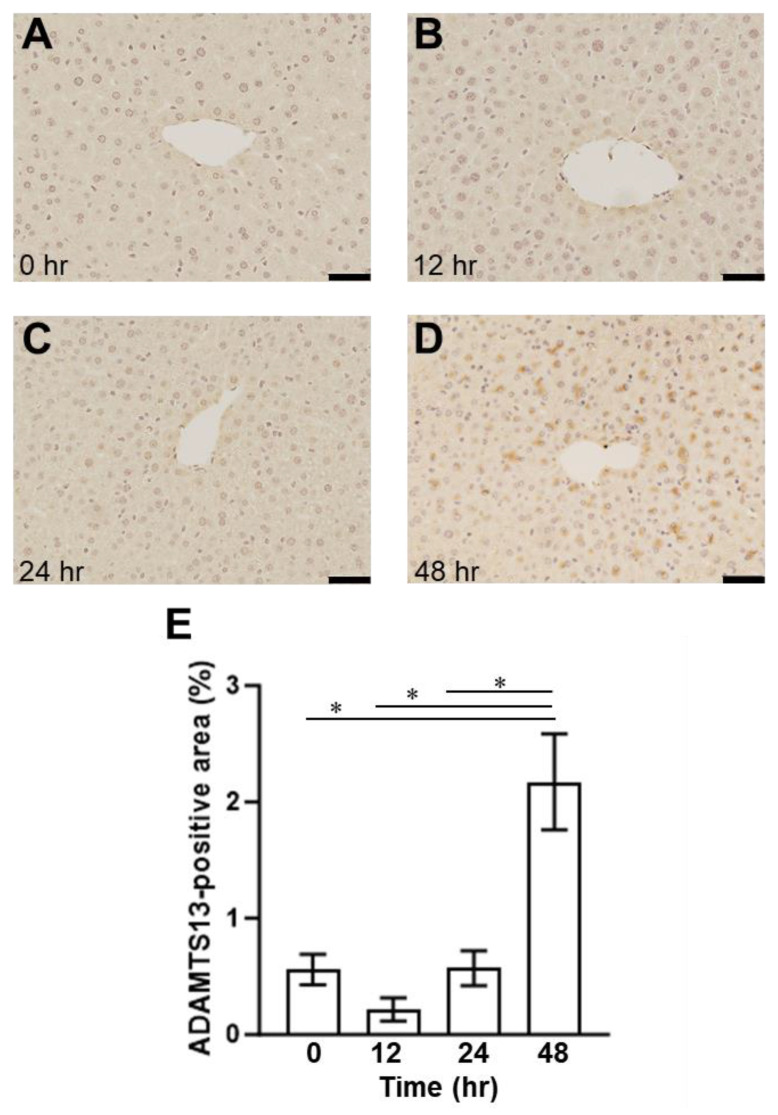
The immunohistochemical findings of ADAMTS13-positive staining in the liver (**A**) 0 h, (**B**) 12 h, (**C**) 24 h, and (**D**) 48 h after MCT injection in the mice. Bars indicate 200 μm. (**E**) Quantitative evaluation of the ADAMTS13-positive area. Data are expressed as mean ± SD (*n* = 4). *, *p* < 0.05.

**Figure 9:**
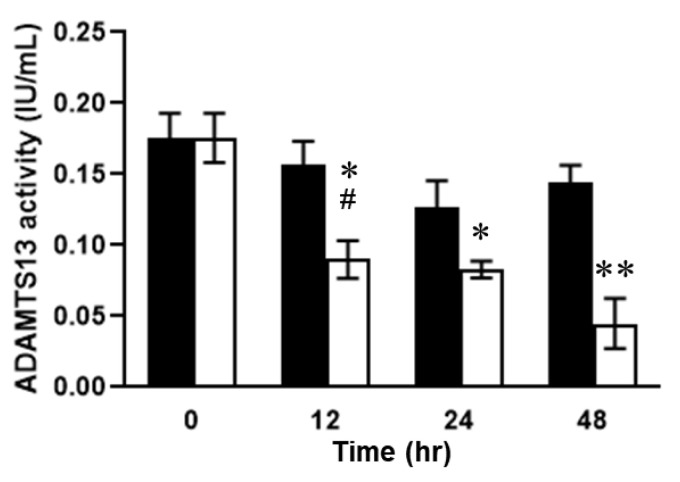
Plasma ADAMTS13 activity (ADAMTS13:AC) levels. Open column, MCT-treated group; closed column, control. Data are expressed as mean ± SD (*n* = 6). *, *p* < 0.01 vs. MCT 0 h; #, *p* < 0.001 vs. control 0 h; ** *p* < 0.01 vs. control 48 h.

**Figure 10:**
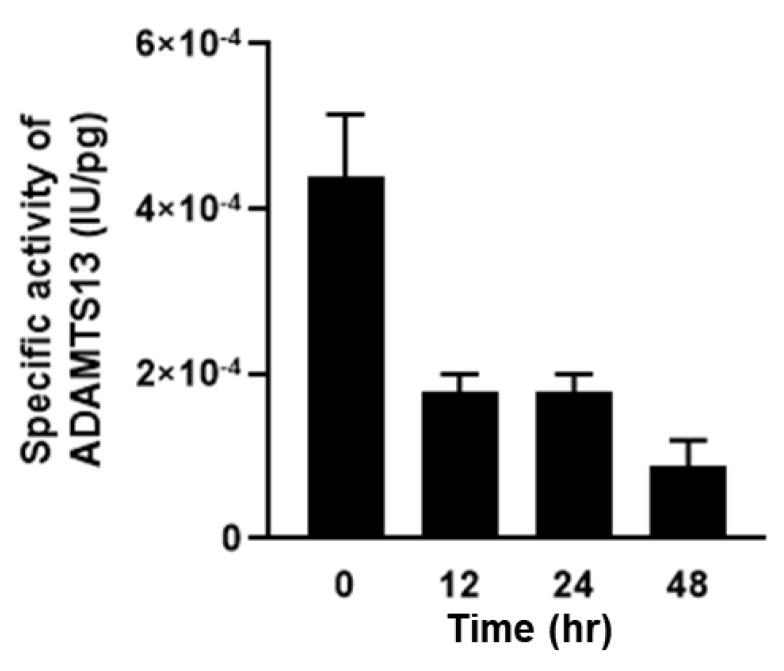
The ratio of ADAMTS13 activity (ADAMTS13:AC) to ADAMTS13 concentrations in MCT-treated groups. Data are expressed as mean ± SD (*n* = 6).

Dynamic distributional changes of ADAMTS13 were then addressed to evaluate the fluctuations in VWF and VWF (A2) levels and the disease severity in the mice with SOS. Furthermore, plasma ADAMTS13 concentrations peaked at 12 h and then decreased in the MCT-treated mice (Figure 7). ADAMTS13 levels were significantly different between the MCT-treated and control groups (Figure 7). In addition, ADAMTS13-positive signals were observed as being enhanced in the livers of mice with SOS using immunohistochemistry (Figure 8). Finally, the enzymatic activity of ADAMTS13 was assayed to determine its functional ability to degrade VWF. The plasma ADAMTS13:AC levels were observed to have declined by 35% at 12 h and were found to be 64% and 30% in the control group mice at 24 h and 48 h, respectively, after MCT administration (Figure 9). The calculated ADAMTS13:AC/concentration ratios clearly demonstrated 61% and 76% decreases in values at 12 h and 48 h after MCT administration, respectively (Figure 10).

## 3. Discussion

SOS has been reported to occur after chemotherapy, pretreatment for blood stem cell transplantation in pediatric hematological malignancies, and liver transplantation and oxaliplatin administration for colorectal cancer liver metastases in the field of gastrointestinal surgery [26,27]. It has a poor prognosis, characterized by hepatocellular necrosis and fibrosis around the lobar sinusoidal endothelium (zone 3), which progresses to portal hypertension and liver failure [28]. Regarding SOS pathogenesis, detachment and desquamation might be observed in sinus endothelial cells [29]. Furthermore, platelets could enter the extravascular Disse lumen and flow toward zone 3 in SOS. Consequently, serotonin and thromboxane A2 would be released from many platelets aggregated in the Disse cavity near zone 3, and the contraction of the central vein gradually continues [30]. Although SOS pathogenesis has been mainly based on liver tissue congestion, we have confirmed extravascular platelet aggregation in the absence of congestion and considered it to be an important early lesion [22].

Platelets contain many growth factors that are required for tissue regeneration or repair. Platelets play a crucial role in promoting liver regeneration and have a preventive effect on the progression of liver fibrosis [31]. In cancer cells, platelets have been shown to stimulate growth in various types of cancer cells, including hepatocellular carcinoma [31]. However, there are contradictory reports that platelets have harmful effects on liver fibrosis and acute liver injury, including viral hepatitis and ischemia-reperfusion injury [31]. Thus, the precise roles of platelets in chronic liver disease and acute liver injury still remain controversial at this time.

VWF is mainly synthesized in vascular endothelial cells and bone marrow megakaryocytes. Moreover, VWF is released when platelets are activated or damaged and forms bonds with membrane proteins on the surface of platelets to promote platelet thrombus formation [15].

The increased platelet aggregation and formation of thrombi promotes microvascular occlusion in the liver and the necrosis of liver tissues. The present study observed increased platelet aggregation outside the blood vessels (Figure 3). Similarly, VWF was also observed in the extravascular space (Figure 4).

In addition, immunostaining showed punctate ADAMTS13 expression outside the blood vessels (Figure 8), demonstrating the histological relationship between ADAMTS13 and VWF in SOS. In addition, the plasma ADAMTS13 concentration significantly increased after 12 h but decreased after 24 h (Figure 7). We hypothesized that the causes of decreased ADAMTS13:AC may include consumption for VWF antigen processing, impaired production and secretion from hepatic stellate cells, and the presence of ADAMTS13 inhibitors [32]. In hepatic cirrhosis, impaired ADAMTS13 production in hepatic stellate cells is thought to cause reduced ADAMTS13:AC [33]. However, ADAMTS13 staining was observed outside the vessel after 48 h in this study (Figure 9). This suggests that impaired ADAMTS13 secretion into the bloodstream may occur in the later stage of SOS; however, in the earlier stage of the disease, other factors may cause a decrease in ADAMTS13:AC. In addition, the increase in VWF was not significantly different after 12 h (Figure 6), suggesting that another factor also affects the decreased ADAMTS13:AC due to ADAMTS13 consumption by VWF antigens.

When the vessel wall is damaged, platelet aggregation and VWF deposition occur locally, and then ADAMTS13 molecules would be consumed. This may be responsible for SOS pathogenesis. Fresh frozen plasma (FFP) administration has been reported to prevent and improve SOS by settling the imbalance of VWF:Ag/ADAMTS13:AC [24]. We also believe that phosphodiesterase (PDE)-III inhibitors and recombinant thrombomodulin (TM), which our laboratory has advocated, are also well suited to preventing SOS [23,34].

A substantial increase in plasma VWF levels with the progression of liver diseases has been reported previously [35]. This is probably due to endothelial damage to the hepatic sinusoid caused by endotoxins and cytokines [22,23,24,29,30,34]. Hepatic cell necrosis, subsequent liver regeneration, and/or high shear stress due to portal hypertension in cirrhotic livers may play major roles in upregulating VWF in the hepatic sinusoidal endothelium. The mechanism responsible for the decrease in ADAMTS13:AC in patients with advanced cirrhosis may include enhanced consumption due to the degradation of large quantities of VWF [36], inflammatory cytokines [37], and/or ADAMTS13 plasma inhibitors [38]. These findings in patients with liver cirrhosis suggest that an imbalance between ADAMTS13 and UL-VWF can induce liver dysfunction due to microcirculatory disturbance [39].

This study suggests that the ADAMTS13:AC/concentration ratio reflects the pathophysiology of early SOS and may be a biomarker for therapeutic interventions. In addition, a further understanding of SOS pathogenesis could be gained if the cause of decreased ADAMTS13:AC were determined from the same perspective. This is the first study to focus on ADAMTS13 activity and concentration in liver diseases including SOS.

## 4. Materials and Methods

### 4.1. Reagents

MCT is a Crotalaria alkaloid extracted from Crotalaria spectabilis at approximately a 3.2% rate. In this study, MCT was purchased from Wako Pure Chemical Industries (Osaka, Japan) and administered to mice at a dose of 270 mg/kg body weight. The MCT was dissolved in 1.0 N hydrochloric acid, and the solution’s pH was adjusted to 7.4 using 1.0 N sodium hydroxide, while the pH was measured using a pH meter. It was then adjusted using phosphate-buffered saline (PBS) to obtain a total volume of 10 mL. The containers were stored at 4 °C and shaded from light.

### 4.2. Animals

D1 mice (females; age: 6–8 weeks; weight: 20–25 g; Charles River, Kanagawa, Japan) were purchased and were allowed free access to water and standard laboratory chow. This study complied with the guidelines of the Division for Animal Research Resources at the University of Kanazawa. Furthermore, all the experiments and procedures were approved by the Animal Care and Use Committee of the University of Kanazawa.

### 4.3. Experimental Protocols

The mice were randomly divided into two groups: MCT-treated and control groups. They were moved to a laboratory animal breeding facility and acclimated to their new environment for 1 week. First, mice were given water and a laboratory diet and fasted for 12 h prior to MCT (270 mg/kg) and PBS administration in the MCT and control groups, respectively. Then, the mice were sacrificed at 0, 12, 24, and 48 h after MCT or PBS administration, and liver tissue and blood samples were collected.

### 4.4. Hematoxylin and Eosin (H&E) Staining and Immunohistochemistry

H&E staining and immunostaining tissue samples were fixed in 4% paraformaldehyde in PBS for 3 days and embedded in a solution of the optimal cutting temperature compound (Sakura Finetek, Tokyo, Japan). Serial 4 μm sections were stained with H&E for histological examination via light microscopy. H&E staining was performed as in our previous study (5–7). For the assessment of EPA, immunostaining was performed using anti-cluster of differentiation 42b (CD42b) (1:100, ab183345, Abcam, Tokyo, Japan). For assessment of aggregating VWF and ADAMTS13 in liver tissue, immunostaining was performed with anti-von Willebrand Factor (1:200, AB7356, Merck Millipore, Rahway, NJ, USA) and anti-ADAMTS13 (1:100, bs 5856R, Bioss Antibodies, Woburn, MA, USA). Each staining was measured for the area stained (%) as the average of four randomly selected images using Image J 1.54a (National Institutes of Health, Bethesda, MD, USA).

### 4.5. Enzyme-Linked Immunosorbent Assay (ELISA)

The levels of ADAMTS13 (ELISA Kit for Von Willebrand Factor Cleaving Protease, SEA950Mu, Cloud-Clone Corp., Katy, TX, USA) and VWF (ELISA for Von Willebrand Factor, CEA833Mu: Cloud-Clone Corp., Katy, TX, USA) in the plasma were measured. In addition, ADAMTS13:AC (ADAMTS13-act ELISA, CY-6000:KAINOS, Tokyo, Japan; the measurement range from 0.005 to 1.00 IU/mL) was assessed using a solid-phase sandwich method with a monoclonal antibody that specifically recognizes the amino acid Tyr1605 at the VWF-A2 (Mouse Von Willebrand Factor A2 ELISA Kit, ab208980, Abcam, Tokyo, Japan) break, which is cleaved by ADAMTS13.

After the blood sample was collected in a tube containing sodium citrate, it was separated into different components via centrifugation. After this, the blood cell and plasma components were extracted from the blood.

### 4.6. Statistical Analysis

Results are expressed as the mean ± standard deviation (SD), and Student’s *t*-test was used to evaluate the differences between the groups.

## 5. Conclusions

Sinusoidal obstruction syndrome (SOS), previously known as veno-occlusive disease (VOD), is a distinctive and potentially fatal form of hepatic injury that occurs after liver transplantation, hematopoietic stem cell transplantation, and the administration of anticancer drugs. Our findings demonstrate that the blood ADAMTS13 activity/concentration ratio will be a useful early biomarker for decision making at the initiation of therapeutic interventions for SOS. Clinical studies are warranted to evaluate this issue further. This blood biomarker may make a significant contribution to survival in patients with SOS in the future.

## Data Availability

The data in this study are available from the corresponding author upon request.
